# Anxiety, Stress Coping Styles and Hope for Success among Graduate Students and High School Graduates during the COVID-19 Pandemic: The Moderating Role of Remote Learning

**DOI:** 10.3390/ijerph19159692

**Published:** 2022-08-06

**Authors:** Sylwia Michałowska, Karolina Rachubińska, Krystian Konieczny

**Affiliations:** Department of Clinical Psychology and Psychoprophylaxis, Institute of Psychology, University of Szczecin, 70-540 Szczecin, Poland

**Keywords:** COVID-19 pandemic, distance learning, mental health

## Abstract

The COVID-19 pandemic has significantly influenced the area of education, in terms of both teaching and learning effectiveness. The aim of the study was to compare groups of high school graduates and graduate students. The Coping Inventory in Stressful Situations (CISS), State and Trait Anxiety Inventory (STAI), Hope for Success Questionnaire (KNS), and our own questionnaire were used in the study. The results of the research show that the group of high school graduates, compared to the graduate students, was characterized by a higher level of anxiety, a style focused on emotions and a lower level of hope for success. People who passed the exam in the online form were characterized by a higher level of anxiety compared to the respondents who passed the exam in the stationary form. According to the results of this study, it can be concluded that the styles of coping and the level of hope for success may be both protective and risk factors related to the level of anxiety during distance learning.

## 1. Introduction

COVID-19 is caused by the highly contagious SARS-CoV-2 virus [1], and due to widespread worldwide transmission and high risk of infection, it was declared a pandemic by the World Health Organization (WHO) [2]. The COVID-19 pandemic started in 2019. Its effects were noticeable shortly after the first cases of the disease spread worldwide. The consequences of this situation are still noticeable in many aspects of people’s lifestyles [3]. The pandemic emergence has had a significant impact on the social, educational, economic, family, and emotional functioning of people around the world. Modified lifestyles based on introduced changes (e.g., self-isolation, quarantine, social distance, distance education) or adopted attitudes (e.g., conspiracy theories or misinformation) have led to a psychosocial crisis [4]. Previous studies have shown an association between SARS (severe acute respiratory syndrome) and mental health problems, including the experience of depression, anxiety, panic attacks, or psychotic symptoms [5].

The area of education—both teaching and learning—had to quickly adapt to the new conditions and requirements. At the beginning of the pandemic, all educational centres, regardless of the educational stage, started the process of distance learning, which led to difficulties such as the lack of appropriate equipment and limited contact with other students and teachers [6].

Pupils and students, at various stages of their education, were forced to change their current ways of functioning, learning, and maintaining relationships. It was associated with changes in the mental functioning of these people, e.g., in terms of anxiety, stress and others [7].

### 1.1. Anxiety

Anxiety is a natural and innate sensation or reaction that can be observed in both animals and humans [8]. It is related to the functioning of the nervous system; therefore, it manifests itself, among other symptoms, through physiological reactions. It can be observed by body tremors, sweating, increased heart rate and blood pressure, and increased muscle tone. The psychological aspect of anxiety is visible in the behaviour, emotions, and cognitive functioning of the person experiencing it. It can also become pathological. This happens when it is not appropriate for the situation, or its intensity is too high [9].

One way to understand anxiety is to distinguish between trait anxiety and state anxiety. This understanding of fear is one-dimensional and was developed with a psychometric approach. One of the classic representatives of this understanding of fear is Spielberger [10]. He defined trait anxiety as an individual predisposition to manifest a specific reaction. On the other hand, state anxiety is defined as physiological arousal and the accompanying conscious feeling of tension, agitation, and emotions related to anxiety [11].

Research shows that the COVID-19 pandemic and its global ramifications are linked to mental deterioration, including increased anxiety. This is particularly related to the age of the respondents—the adverse consequences of the pandemic were greater in the younger part of the study group [12]. The research conducted on students does not indicate a clear increase in the level of anxiety in connection with the implementation of distance learning [13]. The resulting phenomenon of distance learning and its consequences seem to have a multifactorial nature and a diversified impact on students in terms of the type of learning undertaken, effects, and areas of education [14,15,16,17].

The association of the pandemic experience with anxiety symptoms has been widely studied. Among others, it has been shown that in a difficult situation resulting from isolation, emotion-oriented coping strategies and mental withdrawal are associated with higher levels of pandemic anxiety [18]. Additionally, anxiety and insecurity may increase due to a specific situation, such as a child’s disease [19] or pregnancy [20]. At the same time, some study reports indicate that receiving social support may alleviate anxiety associated with the pandemic situation [21].

### 1.2. Stress Coping Styles

Dealing with stress, according to Lazarus and Folkman, has been defined as constantly changing cognitive and behavioural efforts aimed at managing internal and external requirements of the environment that exceed the skills and resources of the individual [22,23]. The three main styles of coping with stress mentioned by the authors of the theory are: the problem-focused coping style, the emotion-focused coping style, and the avoidance-focused coping style [24]. The problem-focused style is characterised as an attempt to change or manage an existing problem that is causing stress, such as involvement in the problem or positive reinterpretation [25]. The style focused on emotions is characterised by an attempt to reduce the level of negative emotions related to a stressful stimulus [26]. The last style of coping with stress distinguished by Lazarus and Folkman—focused on avoidance—can describe the behavioural and cognitive efforts of an individual to minimise and deny stressful situations [23].

Styles of coping with stress are significantly related to the level of anxiety [27], the sense of self-efficacy [28], and the hope for success [29]. Anxiety is an emotion that appears during both long and short-term stress, and at the same time, it can be one of the body’s reactions to an upcoming stressful situation [30]. Other authors have shown that people with low levels of anxiety prefer problem-focused behaviour as a stress-coping style [31]. Research on the level of hope for success and styles of coping with stress indicate a significant relationship between the variables. People with a high level of hope for success are characterised by a style focused on the problem, while a low level of hope for success is associated with the avoidance style [31,32].

During the COVID-19 pandemic, restrictions, mandatory quarantines, and isolation have led to increased tension levels [33]. It has also been shown that people who spent more than 10 days in quarantine were more likely to report symptoms indicating post-traumatic stress disorder [33]. The study by Babicka-Wirkus et al. [34] indicates that maladaptive stress coping strategies used by students, especially during the pandemic, may result in long-term consequences not only for their psychophysiological health but also for their academic performance.

### 1.3. Hope for Success

Hope for success, understood in accordance with the concept created by Snyder, is a construct reflecting a motivational state in which beliefs about having a strong will and the ability to solve emerging problems are active [32,35]. In this approach, an individual hoping for success is convinced that they will be able to achieve the set goal, regardless of whether there are difficulties along the way or experiences of personal doubts, and that in the event of obstacles and difficulties, they will be able to find effective solutions [36].

The concept of hope is important in the context of various psychological states that organise the functioning of an individual, both in a personal and social dimension. Higher hope is associated with the pursuit of better academic achievement [37] or the ability to find benefits in experiencing misfortune [38]. A high level of hope for success is conducive to taking active measures, leads to better adaptation, increases flexibility in stressful situations, and is revealed in social competencies, leading to better family relationships [38]. It is also significantly related to action by bringing the individual closer or further away from the target [39]. Moreover, a person with a high level of hope for success usually presents a lower level of anxiety due to the belief in the ability to find solutions in difficult situations [35,40]. At the same time, a high level of anxiety, through its relationship with the activation of certain beliefs, may contribute to lowering the level of hope for success [41].

Individuals who undertake specific action-oriented activities in the face of stressful situations usually show a high level of hope for success, which is in contrast to people who are characterised by an avoidance style and who usually show a low level of hope for success [32,40].

The pandemic, which is a stressor itself due to its specific conditions, has particularly affected those whose activities have been fully suspended due to virus transmission or whose character has changed significantly, which is also related to the experience of the hope of success. Such groups include sports persons [42,43,44] and schoolchildren [45,46,47,48], as they were fully isolated from their companions in major activities, and their mode of functioning changed dramatically.

Contemporary studies have shown positive correlations between levels of hope for success and life satisfaction [49], psychological well-being, and an optimistic attitude [50].

### 1.4. Distance Learning

Distance learning is a teaching method characterised by the separation of the teacher from the student or group of students, conducted using information technology [51]. This particular type of teaching has been commonly used in connection with the SARS-CoV-2 pandemic, which led to the introduction of distance learning at all educational stages in the summer semester of the 2019/2020 school/academic year. So far, it has been shown that the students’ declared satisfaction with a mode of learning is influenced by a number of factors related not only to the mode but also to the form of adopted interactions, which include the relevance of the content taught or the interactions taking place between a teacher and a student and the quality of this contact, as well as psychological aspects, such as the students’ assessed sense of self-efficacy [52,53]. In spite of many opportunities provided by online learning, there are also important limitations, such as a risk of an increased information gap, especially among students who showed educational deficiencies during classroom learning [54]. Particular attention should be paid to the circumstances in which students in a secondary school graduating class or master’s students preparing for their final examinations must do distance learning without direct contact with a teacher or supervisor and sometimes must also take exams online. The study by Elsalem et al. [55] showed that taking examinations online was associated with greater stress, due to, among other things, concerns about possible technical problems, the quality of the internet connection, or fears about the dishonesty of other students.

### 1.5. Purpose of the Study

The aim of the study was to assess the level of anxiety, stress coping styles, and hope for success among graduate students and high school graduates taking final exams during the COVID-19 pandemic, taking into account the moderating role of distance learning. In their analyses, the authors took into account the learning mode.

### 1.6. Hypotheses

Based on the purpose of the study, the following research hypotheses were formulated:

**H1.**  *There are differences in the level of severity of trait anxiety and state anxiety depending on the type of exam taken (matura exam, master’s exam)*.

**H2.**  *There are differences in the preferred stress coping style, depending on the type of exam taken*.

**H3.**  *There are differences in the level of hope for success, depending on the type of exam taken*.

**H4.**  *There are differences in the level of trait anxiety and state anxiety, depending on the mode of taking the exam (stationary mode, online mode)*.

**H5.**  *There are differences in the preferred stress coping style, depending on the mode of taking the exam*.

**H6.**  *There are differences in the level of hope for success, depending on the mode of taking the exam*.

**H7.**  *There is a relationship between the levels of trait and state anxiety and distance learning performance assessment*.

**H8.**  *There is a relationship between the preferred stress coping style and distance learning performance assessment*.

**H9.**  *There is a relationship between the level of hope for success and distance learning performance assessment*.

**H10.**  *There is a relationship between the levels of trait and state anxiety and the preferred stress coping style*.

**H11.**  *There is a relationship between the level of trait and state anxiety and the level of hope for success*.

**H12.**  *There is a relationship between the preferred stress coping style and the level of hope for success*.

## 2. Material and Methods

### 2.1. Research Tools

For the purposes of the analyses described, a battery consisting of an original survey was created, which included the Coping Inventory in Stressful Situations (CISS) in the Polish adaptation of P. Szczepaniak, J. Strelau, and K. Wrześniewski, the State–Trait Anxiety Inventory (STAI) in the Polish adaptation of C.D. Spielberger, J. Strelau, M. Tysarczyk, K. Wrześniewski, and the Questionnaire of Hope for Success (KNS) by M. Łaguna, J. Trzebiński, and M. Zięba. The research methods were chosen based on the analysis of the literature and previous studies on students in a secondary school graduating class or master’s students who took their final examinations in the traditional mode prior to the pandemic and related changes in the form of hybrid teaching or new distance learning methods. All of the selected instruments have documented relevance, reliability, and up-to-date standards and have been successfully used in research on the age group adopted in this publication.

The Coping Inventory for Stressful Situations (CISS) Questionnaire by N.S. Endler and J.D.A. Parker is an instrument for measuring behaviours typical of people in distress. These behaviours are categorised according to three scales: task-oriented coping (TOC), emotion-oriented coping (EOC), and avoidance-oriented coping (AOC), which may take two forms: involvement in displacement activity (IDA) and search for social contacts (SSC). The CISS consists of 48 statements, and the study subject is asked to give answers on a 5-point Likert scale, which relates to the frequency of taking a specific action in situations that are stressful for a person. The instrument has high internal consistency regarding individual scales (0.78–0.90) and satisfactory stability (the correlation coefficient between two surveys at an interval of 2–3 weeks was in the range of 0.73–0.80). Factor validity was also demonstrated for the instrument [56].

The State–Trait Anxiety Inventory (STAI) by C.D. Spielberger, R.L. Goruch, and R.E. Lushene is used to measure two dimensions of anxiety: anxiety as a personality trait, which is relatively stable, and anxiety understood as a transient state, which is situationally conditioned. Therefore, the STAI consists of two subscales one for trait anxiety and the other for state anxiety. Each subscale contains 20 items, and the study subject answers them by ticking one of four responses (“definitely not”, “rather not”, “rather yes”, “definitely yes”). The instrument has high internal consistency (state anxiety: from 0.89 to 0.92, trait anxiety: from 0.76 to 0.90) [57].

Another instrument used in the study was the Hope for Success Questionnaire (Pol. Kwestionariusz Nadziei na Sukces, KNS) by M. Laguna, J. Trzebinski, and M. Zięba. The instrument consists of 12 statements, whose applicability is rated by the study subject on an 8-point scale (from “definitely untrue”—1 to “definitely true”—8). The KNS measures their hope for success, understood as the strength of one’s expectation for a positive outcome of one’s actions. It includes two main components: the belief in having a strong will and the belief in their ability to find solutions. The questionnaire has satisfactory internal consistency [35].

The proprietary questionnaire used in the study consisted of 14 questions covering several information areas, including information of a sociodemographic nature, such as age, gender, place of residence, and voivodeship. The survey made it possible to collect individual information about the educational stage of the respondents, including the profile of the school, class profile or field of study, the type of exam taken, and the date and mode of the exam being taken. The respondents assessed the effectiveness of remote learning from insufficient to very good and determined whether, to what extent (significant, moderate, slight, or none), and in what way (helped, hindered), in their opinion, remote learning had an impact on their ability to prepare for the exam. The ethics committee approved the study plan and gave its consent to conduct it.

### 2.2. Procedure

The study used purposeful selection, limiting the composition of the study sample only to people who took the *matura* exam or graduate exams in the second semester of the 2019/2020 school/academic year. Before starting the procedure, the participants were always informed about the full voluntary participation and the possibility of withdrawing from participation in the study at any time. The respondents were assured of the full confidentiality of the research procedure and the use of the results only for scientific purposes. The respondents gave their voluntary consent to participate in the procedure without receiving any remuneration for it. Due to the state of the pandemic, the entire procedure was conducted online, collecting data using the Google Forms service. The respondents were invited to participate in the study by sharing information about it on social media. The data collection procedure started on 5 May 2020 and ended on 5 June 2020, obtaining a satisfactory sample of respondents.

### 2.3. Group

A total of 291 people started to fill out the test battery, 56 of whom did not answer all the questions, and the final analyses were performed using the data collected from 235 participants. Out of all participants, 126 people were high school graduates, and 106 people were graduate students.

The majority (72.6%) of the respondents were women. The respondents were between the ages of 18 and 40 years (SD = 3.27, M = 21). The most numerous group consisted of people aged 19 (25.4%). Of the respondents, 5 (2.1%) were between 28 and 40 years of age. The participants in the study lived mainly in rural areas (31%) or very large cities with more than 500,000 inhabitants (26.9%).

Pupils and students from all 16 voivodeships participated in the study; the most respondents (15.2%) came from the Lower Silesian Voivodeship and the least (0.5%) came from the Lubusz Voivodeship. The respondents represented almost all types of schools and universities to a different extent. The lowest number of respondents was from the Academy of Arts (1%) and the highest number from a university (35%).

### 2.4. Data Analysis

Statistical analyses were performed using IBM SPSS Statistics 25.0. The program was used to calculate the basic measures of descriptive statistics together with the test of the normality of the distribution. In order to establish the relationship between the variables, a Pearson or Spearman correlation analysis was performed. In order to compare the two groups in terms of quantitative variables, a t-test analysis was performed for independent samples, and when more groups were compared, a one-way analysis of variance was conducted. In order to establish the relationship between the categorical variables, the analysis was performed with the Pearson χ^2^ test or with Fisher’s exact test, if the expected number was less than 5. The level of significance for the analysis was assumed to be α = 0.05.

## 3. Results

Analyses were performed to compare the groups of high school graduates and graduate students in terms of coping styles, hope for success, and anxiety as a trait and as a state. A t-test analysis was carried out for independent samples. The analysis showed significant differences between the groups in terms of the level of anxiety as a state and trait, task-focused style (SSZ), emotion-focused style (SSE), the search for a social angle (PKT) account, and the level of hope for success. In the group of high school graduates, there was a significantly higher level of anxiety as a state, and as a trait, a higher intensity of the style focused on emotions and a higher hope for success compared to the group of graduate students. The graduates, on the other hand, displayed a higher level of task-focused style and a higher level of searching for social contacts as a style of coping with stress. The strength of the effect of the differences was low to moderate. The groups did not differ in terms of emotion-centred style, involvement in surrogate activities, solution-finding skills, or willpower. The results of the analyses are presented in Table 1.

In order to compare the levels of anxiety, coping styles, and hope for success in the group of graduate students due to the mode of the exam for which they were preparing (in-person or online), a *t*-test analysis was carried out for independent samples.

The analysis showed that the graduate students who were preparing for the online exam displayed a higher level of state anxiety, a higher intensity of the emotion-focused style, and a lower intensity of the style of coping with stress focused on avoidance, including seeking social contacts. The strength of the effect on the differences was weak to moderate. The results of the analyses are summarised in Table 2.

In order to establish the relationship between the assessment of learning effectiveness and the levels of anxiety, coping styles, and hope for success, a Spearman correlation analysis was performed. The analysis showed that the higher the levels of state anxiety, trait anxiety, and emotional style were, the lower the e-learning effectiveness was (weak and negative correlations). A positive and weak relationship was noted between the assessment of learning effectiveness and the search for social contacts as a style of coping with stress. This means that the higher the intensity of this style, the higher the assessment of the effectiveness of e-learning was. There were no significant relationships between the assessment of the effectiveness of e-learning and the style of coping with task-focused coping, avoidance, engaging in substitute activities, or the hope for success and its dimensions. The results of the analyses are presented in Table 3.

Pearson’s correlation was used to investigate the relationships between the levels of anxiety, coping styles, and the hope for success. A detailed analysis of the results showed that anxiety as a state was strongly and positively associated with the style focused on avoidance and weakly and positively associated with engaging in substitute activities. A negative correlation at a low to moderate level was found with the task-focused style, social seeking, solution-finding skills, and willpower. This result suggests that the higher the level of state anxiety is, the higher the level of avoidance-focused style, the higher the level of involvement in substitute activities, the lower the level of task-focused coping and social seeking, and the lower the ability to find solutions and willpower. Trait anxiety was negatively correlated at low to moderate levels with a task-focused style, hope for success, the ability to find solutions, and willpower. Positive relationships were noted with the style focused on emotions (strong correlation) and avoidance, including engaging in substitute activities (weak correlations). The avoidance-focused style was positively correlated at the moderate and strong levels with the hope for success, the ability to find solutions, and willpower—the higher the severity of this style, the higher the level of hope for success, the ability to find solutions and willpower. The emotion-focused style was negatively and moderately correlated with the ability to find solutions and willpower. In turn, the styles of coping focused on avoidance and engaging in substitute activities were weakly and negatively related to the ability to find solutions. Engaging in surrogate activities was also negatively and weakly related to willpower, and seeking social contact was positively and weakly related to willpower. Detailed results of the analyses are presented in Table 4.

## 4. Discussion

Distance learning can be significantly related to the level of anxiety [13,15], styles of coping with stress [52], and the level of hope for success. During the SARS-CoV-2 coronavirus pandemic, people taking the *matura* exam and completing their master’s degree had to adapt their skills and educational requirements to the rapidly changing epidemiological situation. In many schools, teaching did not take place in real time, and students had to rely heavily on self-directed learning skills [58]. Among the tools used for teaching during a pandemic, the following were distinguished: materials sent by e-mail, materials sent to the platform of the educational unit, chat, online classes, and activities on other platforms. The results of the study showed that the most effective form of distance learning in terms of learning efficiency in the group of graduate students was e-mail, while among high school graduates, the materials sent to the educational unit’s platform were the most effective. The high school graduates believed that this form of distance learning would allow them to prepare for the *matura* exam most effectively. Further statistical analyses showed that the higher the levels of anxiety as a state and anxiety as a trait, the lower the evaluation of the effectiveness of distance learning. Research by other authors also highlights the importance of remote learning tools and the type of remote learning activities. A remote lecturer has been shown to be more effective than other forms of classes, such as seminars. This is related to the amount of interaction between the lecturer and students [59].

It was also noticed that high school graduates, compared to graduate students, were characterised by higher levels of anxiety as a state and anxiety as a trait and a preference for a style focused on emotions. The results of our research are consistent with the research of other authors who found that high school graduates showed a style focused on emotions and a high level of anxiety [31]. The studies by Aysan, Thompson, and Hamarat [60] and Shaikh et al. [61] indicate a relationship between a preference for more effective coping styles and an increase in age, e.g., focused on the task. These results are confirmed by the authors of this study; graduates compared to high school graduates more often chose the style focused on the task and avoidance. Further analyses also showed that the level of hope for success was significantly higher in the group of high school graduates. These results contradict the results of other authors who have assumed that the level of hope for success was higher among people preferring the task-focused style of coping with stress [31,32,35].

Examination anxiety is the most visible source of anxiety among students [62]. The SARS-CoV-2 coronavirus pandemic has resulted in many graduate exams being held online. The analyses indicate a relationship between online exams and a higher level of anxiety in respondents compared to exams conducted in a traditional form. This is probably related to the previously unknown form of the exam and difficult communication with supervisors. Research by other authors has also shown a high level of anxiety among students related to online examinations [63] and a reluctance to participate in online classes [64]. Arora et al. [63] also emphasised that the level of anxiety associated with passing the online exam was higher than that associated with the onset of the pandemic.

## 5. Conclusions

The period of the SARS-CoV-2 coronavirus pandemic has been a challenge for the world for many reasons. Difficulties have also been encountered by people taking *matura* and graduate exams. The method of conducting online classes and the type of tool used by the school may be related to the assessment of the effectiveness of distance learning and the level of preparation for the exam. It is important from the point of view of the developing e-learning system in the education system. According to the results of the above studies, it can be concluded that the styles of coping with stress can be both a protective factor and a risk factor related to the level of anxiety during distance learning. Graduates who preferred the style focused on the task showed a lower level of anxiety both as a state and as a trait compared to high school graduates who preferred the emotion-focused style.

The individual characteristics of pupils and students turned out to be an important factor related to the functioning and process of education during the pandemic. New, functional technological solutions in the field of teaching made it possible to continue education, despite many limitations related to everyday functioning.

It was proven that in comparison to master’s students, students in a secondary school graduating class were characterised by higher levels of state anxiety and trait anxiety and that they preferred an emotion-oriented coping style. In comparison to students in a secondary school graduating class, master’s students were more likely to use task-oriented and avoidance-oriented coping styles. The higher the levels of state anxiety and trait anxiety in the respondents, the lower their assessment was of the effectiveness of the distance learning they had participated in. It seems that the level of anxiety felt by those preparing for final examinations and the coping style that dominates their behaviour may be related to the fact that they differently assess the forms of learning offered to them, and therefore, may use them differently. These findings can therefore be used in the future for the elaboration and implementation of curricula or final exam preparation courses based on individual characteristics of participants to increase their relevance and effectiveness.

The analyses showed an association of online examinations with higher levels of anxiety in study subjects compared to examinations conducted in a stationary, traditional format. This is probably related to an unfamiliar form of the exam and difficult communication with supervisors. Therefore it seems reasonable that in situations where examinations can be taken only remotely, care should be taken to ensure the comfort and emotional security of examinees, e.g., by allowing them to check the technical operation of the equipment in advance or to organise a mock exam. Particularly relevant findings indicate the need to ensure fluency and accessibility of communication with teachers and supervisors for students in a secondary school graduating class and master’s students preparing for their final examinations.

The study also has its limitations, which need to be taken into account in subsequent research in this area. The vast majority of respondents were female (72.6%), which may have affected the results obtained in the study, e.g., in the anxiety measurement. Another limitation of the study may be the large age span of the respondents (18 to 40 years old). The results indicate that a preference for task-oriented coping increases with age. Due to the pandemic situation, the study was conducted in an online format; this should be considered a potential limitation in terms of group selection and the reliability of the obtained results [65].

## Figures and Tables

**Table 1:** Comparison of high school and graduate students in terms of coping styles, hope for success, and anxiety as a state and as a trait.

	High School Graduates (*n* = 129)		University Graduates (*n* = 106)				95% CI		
	M	SD	M	SD	t	*p*	LL	UL	Cohen’s d
State anxiety	48.68	13.22	41.80	10.50	4.45	<0.001	3.83	9.93	0.57
Trait anxiety	49.43	10.64	42.56	8.87	5.40	<0.001	4.36	9.38	0.70
SSZ	54.78	9.69	58.90	7.00	−3.78	<0.001	−6.27	−1.97	0.48
SSE	52.67	11.03	46.55	9.08	4.67	<0.001	3.54	8.71	0.60
SSU	44.00	9.78	44.50	8.31	−0.42	0.677	−2.86	1.86	0.05
ACZ	17.78	4.92	17.32	4.37	0.74	0.459	−0.75	1.66	0.10
PKT	14.98	4.62	16.10	3.55	−2.10	0.037	−2.17	−0.07	0.27
Hope for success	64.50	7.93	61.84	9.52	2.33	0.020	0.41	4.90	0.31
Ability to find solutions	23.93	4.63	23.73	5.40	0.31	0.756	−1.09	1.49	0.04
Willpower	20.12	5.25	21.25	4.38	−1.81	0.071	−2.38	0.10	0.23

SSZ—task-oriented coping (styl skoncentrowany na zadaniu); SSE—emotions-oriented coping (styl skoncentrowany na emocjach); SSU—avoidance-oriented coping (styl skoncentrowany na unikaniu); ACZ—involvement in displacement activity (angażowanie się w czynności zastępcze); PKT—search for social contacts (poszukiwanie kontaktów towarzyskich).

**Table 2:** Comparison of anxiety levels, coping styles, and hope for success among graduate students due to the exam mode.

	Traditional Mode (*n* = 51)		Online Mode (*n* = 55)				95% CI		
	M	SD	M	SD	t	*p*	LL	UL	Cohen’s d
State anxiety	39.57	10.71	43.87	9.95	−2.15	0.034	−8.28	−0.33	0.42
Trait anxiety	41.00	9.55	44.00	8.01	−1.76	0.082	−6.39	0.39	0.34
SSZ	58.08	6.20	59.65	7.64	−1.17	0.245	−4.25	1.10	0.22
SSE	44.61	7.65	48.35	9.97	−2.15	0.034	−7.18	−0.30	0.42
SSU	46.63	6.84	42.53	9.09	2.64	0.010	1.01	7.19	0.51
ACZ	18.08	3.88	16.62	4.70	1.74	0.085	−0.21	3.13	0.34
PKT	17.00	3.28	15.27	3.61	2.57	0.012	0.40	3.06	0.50
Hope for success	61.08	8.72	62.55	10.24	−0.79	0.431	−5.14	2.21	0.15
Ability to find solutions	23.82	5.32	23.64	5.52	0.18	0.859	−1.90	2.28	0.03
Willpower	21.45	3.77	21.07	4.91	0.44	0.659	−1.32	2.07	0.09

SSZ—task-oriented coping (styl skoncentrowany na zadaniu); SSE—emotions-oriented coping (styl skoncentrowany na emocjach); SSU—avoidance-oriented coping (styl skoncentrowany na unikaniu); ACZ—involvement in displacement activity (angażowanie się w czynności zastępcze); PKT—search for social contacts (poszukiwanie kontaktów towarzyskich).

**Table 3:** Spearman’s correlations between the assessment of learning effectiveness and the level of anxiety, coping styles and hope for success.

	Assessment of the Effectiveness of Remote Learning	
	r_s_	*p*
State anxiety	−0.28	<0.001
Trait anxiety	−0.24	<0.001
SSZ	0.09	0.158
SSE	−0.27	<0.001
SSU	0.05	0.442
ACZ	−0.07	0.263
PKT	0.16	0.013
Hope for success	0.01	0.845
Ability to find solutions	0.12	0.070
Willpower	0.12	0.066

SSZ—task-oriented coping (styl skoncentrowany na zadaniu); SSE—emotions-oriented coping (styl skoncentrowany na emocjach); SSU—avoidance-oriented coping (styl skoncentrowany na unikaniu); ACZ—involvement in displacement activity (angażowanie się w czynności zastępcze); PKT—search for social contacts (poszukiwanie kontaktów towarzyskich).

**Table 4:** Pearson’s correlations between anxiety, coping styles, and hope for success.

	1	2	3	4	5	6	7	8	9	10
1. State anxiety	1									
2. Trait anxiety	0.68 **	1								
3. SSZ	−0.32 **	−0.40 **	1							
4. SSE	0.69 **	0.76 **	−0.33 **	1						
5. SSU	0.05	0.13 *	−0.04	0.26 **	1					
6. ACZ	0.17 **	0.26 **	−0.17 **	0.36 **	0.80 **	1				
7. PKT	−0.17 **	−0.10	0.15 *	−0.01	0.75 **	0.27 **	1			
8. Hope for success	−0.08	−0.16 *	0.44 **	−0.07	−0.03	−0.08	0.04	1		
9. Ability to find solutions	−0.34 **	−0.50 **	0.53 **	−0.35 **	−0.18 **	−0.23 **	−0.02	0.78 **	1	
10. Willpower	−0.30 **	−0.48 **	0.50 **	−0.34 **	0.05	−0.19 **	0.29 **	0.69 **	0.54 **	1

** *p* < 0.01; * *p* < 0.05. SSZ—task-oriented coping (styl skoncentrowany na zadaniu); SSE—emotions-oriented coping (styl skoncentrowany na emocjach); SSU—avoidance-oriented coping (styl skoncentrowany na unikaniu); ACZ—involvement in displacement activity (angażowanie się w czynności zastępcze); PKT—search for social contacts (poszukiwanie kontaktów towarzyskich).

## Data Availability

Not applicable.
